# Olfactory three needle regulates the proliferation of olfactory bulb neural stem cells and ameliorates brain injury after subarachnoid hemorrhage by regulating Wnt/β-catenin signaling

**DOI:** 10.1016/j.heliyon.2024.e28551

**Published:** 2024-03-25

**Authors:** Feng Zhou, Zhenzhi Wang, Kang Xiong, Meiling Zhang, Qiang Wang, Yuan Wang, Xiong Li

**Affiliations:** a Department of Neurosurgery, The Affiliated Hospital of Shaanxi University of Chinese Medicine, Xianyang, Shaanxi, 712020, China; b Department of Chinese and Western Medicine, Shaanxi University of Chinese Medicine, Xianyang, Shaanxi, 712046, China; c Combination of Acupuncture and Medicine Innovation Research Center, Shaanxi University of Chinese Medicine, Xianyang, Shaanxi, 712046, China

**Keywords:** Subarachnoid hemorrhage, Olfactory three needle, Neuronal damage, Neural stem cell differentiation, Wnt/β-catenin signaling

## Abstract

**Background:**

Subarachnoid hemorrhage (SAH) is a serious cerebrovascular emergency. The incidence of SAH and hazard ratio of death increase with age.

**Objective:**

In this study, we aimed to observe the effects and potential mechanisms of olfactory three needle (OTN) on cognitive impairment, neuronal activity, and neural stem cell differentiation in SAH rats.

**Methods:**

Sprague-Dawley (SD) rats were randomly divided into five groups: Sham, SAH group, SAH + Nimodipine (NMP) group, and SAH + OTN group. The rats in the SAH + OTN group received the OTN electroacupuncture treatment. For treatment with recombinant DKK1 (a Wnt/β-catenin inhibitor), mice were injected with DKK1.

**Results:**

Our results found that OTN improved cognitive impairment and hippocampal neuron damage in SAH rats. Furthermore, OTN promoted the proliferation of neural stem cells in SAH rats. Mechanistically, OTN activated Wnt/β-catenin signaling in SAH rats, as indicated by the increased expression levels of Wnt1, β-Catenin, LMNB1, and *p*-GSK-3β. DKK1 reversed the improvement effect of OTN on cognitive impairment and neuronal damage in SAH rats. Meanwhile, DKK1 blocked the promoting effect of OTN on the proliferation of NSCs in SAH rats.

**Conclusions:**

OTN electroacupuncture may be an effective therapeutic strategy for SAH.

## 1 Introduction

Aneurysmal subarachnoid hemorrhage (SAH) is due to the blood leaking into the subarachnoid space because of the sudden rupture of cerebral vessels, which accounts for about 7–10% of the whole stroke [1]. Patients with SAH have a very high mortality and disability rate, but there is currently a lack of effective treatment [2]. With the development of surgery and interventional therapy, researchers have had great success in the control of ruptured aneurysms, but the secondary brain injury caused by subarachnoid hemorrhage still lacks effective treatment measures [2,3]. In recent years with the aging of the population, brain injury caused by SAH not only brings serious harm to the patient's own but also brings a heavy economic burden to their family and society [4].

The blood released into the subarachnoid space after SAH and produces a series of complex pathophysiological processes. Currently, SAH is commonly defined in terms of its neurological repercussions including raised intracranial pressure, decreased cerebral perfusion pressure and cerebral blood flow, blood-brain barrier disruption, brain edema, brain swelling, and severe cerebral vasospasm [5,6]. These changes will induce the activation of cytokines and signaling pathways, including oxidative stress, inflammatory reaction, and neuronal apoptosis, resulting in brain damage and poor prognosis [7,8]. Therefore, timely reversal of neuronal apoptosis is an important measure to improve brain dysfunction secondary to SAH.

The discovery of neural stem cells (NSCs) broke the claim that neurons in the central nervous system cannot regenerate and brought new hope for the treatment of SAH [9,10]. NSCs, a type of stem cell, are capable of self-renewal which may be maintained for life-long proliferation and differentiation [11,12]. Accumulating evidence suggests that NSC is characteristic to differentiate into three main kinds of central nervous system cells: neurons, astrocytes, and oligodendrocytes [11]. Thus, NSCs have a repairing effect on damage to the nervous system.

Traditional Chinese medicine (TCM) argues that the action mechanism of SAH can be due to blood stasis [13]. Acupuncture therapy is one of the particular curative methods of Chinese medicine [14]. Because of the advantages of acupuncture in safety, convenience, and economy, the number of stroke patients receiving acupuncture treatment is increasing year by year [15,16]. For the olfactory three needle (OTN), Yingxiang (LI 20) acupoint is the main point, and Yintang (GV 29) point is the auxiliary point, which was reported for the treatment of neurological disorders [17,18]. However, the effect and mechanism of OTN on SAH have rarely been investigated.

In this study, we constructed an animal model of SAH, which was used to receive acupuncture of OTN. We explored the regulatory effect and molecular mechanism of OTN on neuronal survival and NSC proliferation after SAH.

## 2 Methods and materials

### 2.1 Animals

Male Sprague-Dawley (SD) rats were purchased from Chengdu Dossy Experimental Animals CO.,LTD. (Chengdu, Sichuan). The feeding environment was 25 ± 1 °C, relative humidity 50%–60%, and light/darkness for 12 h circulation. Rats are allowed to eat and drink freely. Animals and experimental protocol were conducted according to the guidelines and ethical standards of the Animal Care and Use Ethics Committees of Shaanxi University of Traditional Chinese Medicine (SUCMDL20180313015). The study was carried out in compliance with the ARRIVE guidelines.

### 2.2 SAH model

An experimental SAH model was performed in rats using the blood injection method as previously described [19]. Rats were anesthetized using 4% isoflurane and anesthesia was maintained with 2% isoflurane. The rats were fixed on a surgical splint in the supine position. The rats were intubated and then ventilated with a rodent respirator (683; Harvard Apparatus Inc., USA), which maintained 70/30% medical air/oxygen. A midline scalp incision was made, and a hole was drilled in the anterior fontanelle 2.5 mm, and midline 1.3 mm. The pia mater was punctured under the microscope to expose the subarachnoid space. SAH was produced by injection of 300 μL blood withdrawn from the tail artery, through a 27-gauge spinal needle with a rounded tip and side hole, which was inserted into the suprachiasmatic cistern. Rats in the sham group underwent the same procedures, except for blood injections.

### 2.3 Electroacupuncture treatment

Electroacupuncture OTN point began 1 day before the operation. Use sterile stainless steel needles (Huatuo, Suzhou, China), 0.3 cm oblique puncture at Yingxiang (LI 20) point inward and upward, and 0.3 cm flat puncture at Yintang (GV 29) point towards the root of the nose. Stimulation was then generated with the EA apparatus (G6805-2A, Huayi, Shanghai, China). The stimulation parameters were set as follows: density wave, frequency 80–100 Hz, current intensity 1–3 mA, voltage 1–3 V. Needle-retention time lasted 10 min. This treatment was administered once a day for 7 days.

#### 2.3.1 Experimental design

**Fig. 1:**
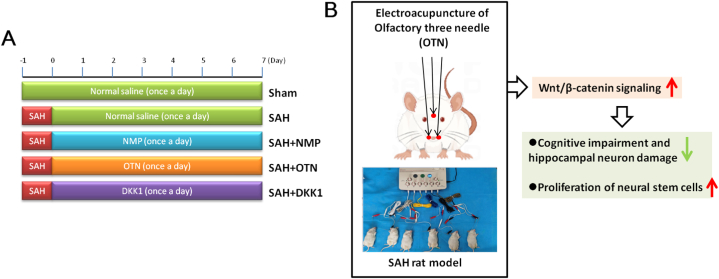
**OTN regulated the proliferation of olfactory bulb neural stem cells and ameliorated brain injury after SAH by regulating Wnt/β-catenin signaling.** (A) The panel displays a graphical representation of the study layout. (B) The panel displays the OTN mechanism diagram.

The experimental design timeline is shown in Fig. 1A.

Experiment 1 Rats were randomly assigned to five groups (n = 6/group): Sham, SAH group, SAH + Nimodipine (NMP) group, and SAH + OTN group. For the SAH + Nimodipine (NMP) group, the rats were given 12 mg/kg Nimodipine (Bayer HealthCare, Beijing, China) orally daily, which was used as positive control drugs. For the SAH + OTN group, the rats received the OTN treatment for 7 consecutive days. Rats in the sham group were given saline by gavage. Sham electroacupuncture superficial skin penetration (1–2 mm in depth) at non-acupoints without electricity was performed in the Sham group.

Experiment 2 Rats were randomly assigned to five groups (n = 6/group): Sham, SAH group, SAH + OTN group, SAH + DKK1 (a Wnt/β-catenin inhibitor) group, SAH + OTN + DKK1 group. For the SAH + OTN group, the rats were treated with OTN once a day for 7 consecutive days. For the SAH + DKK1 group, the rats were injected with DKK1 (ip. 50 μg/day for 7 consecutive days). For the SAH + OTN group, the rats were treated with OTN once a day for 7 consecutive days and were injected with DKK1(ip. 50 μg/day for 7 consecutive days). For the Sham and SAH groups, the rats were injected with an equivalent volume of saline. Sham electroacupuncture superficial skin penetration (1–2 mm in depth) at non-acupoints without electricity was performed in the Sham group.

### 2.4 Morris water maze test

Rats were trained and tested with a Morris water maze in a circular pool of 1.5 m diameter to assess their cognitive and memory abilities as described before [20]. All data were recorded by a camera above the pool. Behavioral indicators of rats were analyzed using a computer connected to a camera.

### 2.5 Hematoxylin and eosin (H&E) stain

The hippocampus of rats was collected and fixed in 4% paraformaldehyde overnight, processed, and embedded in paraffin. The tissue sections were stained with hematoxylin and eosin (H&E) to observe the degree of the lesion and inflammatory cell infiltration under 400 × times magnification optical microscope (Olympus BH2, Tokyo, Japan).

### 2.6 Immunofluorescence (IF) staining

After the experiments, the olfactory bulb and hippocampus were dissected and fixed with 4% paraformaldehyde. Paraffin sections of the olfactory bulb and hippocampus were dewaxed and hydrated. The sections were incubated in QuickBlock™ Blocking Buffer (Beyotime, Shanghai, China) for 30 min at room temperature. Then, the sections were incubated with the anti-MAP2 (Abcam, ab254264; 1/100), anti-GFAP (Abcam, ab4648; 1/100), Nestin (Abcam, ab105389; 1/100, BrdU (Abcam, ab18256; 1/100), or GSK-3β (Cell Signaling Technology, #12456; 1/200), at 4 °C overnight and washed 3 times with phosphate-buffered saline (PBS). The staining of the olfactory bulb and hippocampus was observed under a fluorescence microscope BX53 (Olympus, Tokyo, Japan) at 200× magnification. The acquired images were analyzed using Image-J software (National Institutes of Health, Bethesda, USA) to measure the fluorescence intensity (IntDen) and area. Subsequently, the mean fluorescence intensity for each image was computed.

### 2.7 Western blot analysis

The olfactory bulb and hippocampus tissue lysis solution were fabricated using RIPA buffer (Signaling Technology, Inc.). The protein concentration was examined by a BCA kit (Sigma-Aldrich; Merck KGaA). Total protein (30 μg/sample) was separated via 10% SDS-PAGE and nitrocellulose membranes. Use 5% nonfat dried milk to block the membranes The corresponding protein antibodies were as follows: NSE (Abcam, ab79757; 1/1000), TH (Abcam, ab137869; 1/5000), Choline Acetyltransferase (CHAT, Abcam, ab181023; 1/1000), Glutaminase (GLS, Abcam, ab156876; 1/1000), Ki67 (Abcam, ab1667; 1/1000), proliferating cell nuclear antigen (PCNA, Cell Signaling Technology, #2586; 1/2000), Wnt1 (Abcam, ab15251; 1/1000), β-Catenin (Abcam, ab32572; 1/5000), GSK-3β (Cell Signaling Technology, #12456; 1/1000), *p*-GSK-3β (Cell Signaling Technology, #5558; 1/1000), and β-actin (Boster, BM0627; 1/1000). Then, the membrane washing was performed with Tris-buffered saline/0. 1% Tween (TBST) and incubated for 1.5 h with an HRP Goat anti-Rabbit IgG (Abcam, ab6721). The band visualization was carried out using the ECL system (Affinity Biosciences, Cincinnati, Ohio, USA) and as an internal control, β-actin was used. The bands were detected using the Tanon fluorescent image analysis system software V2.0 (Shanghai, China), and the resulting exposure data were scanned using Gel-Pro analyzer4 software (Atto, Tokyo, Japan) and quantified as the integrated optical density (IOD) of the target protein. Relative protein expression was calculated.

### 2.8 Statistical analysis

Means and standard deviations were used to represent the data. All data were analyzed using SPSS 22.0 software (IBM Corp., Armonk, NY, USA). A one-way ANOVA with Tukey post hoc test of means was used for multiple group comparisons. P < 0.05 was determined as statistically significant.

## 3 Results

### 3.1 OTN improved cognitive impairment and hippocampal neuron damage in SAH rats

**Fig. 2:**
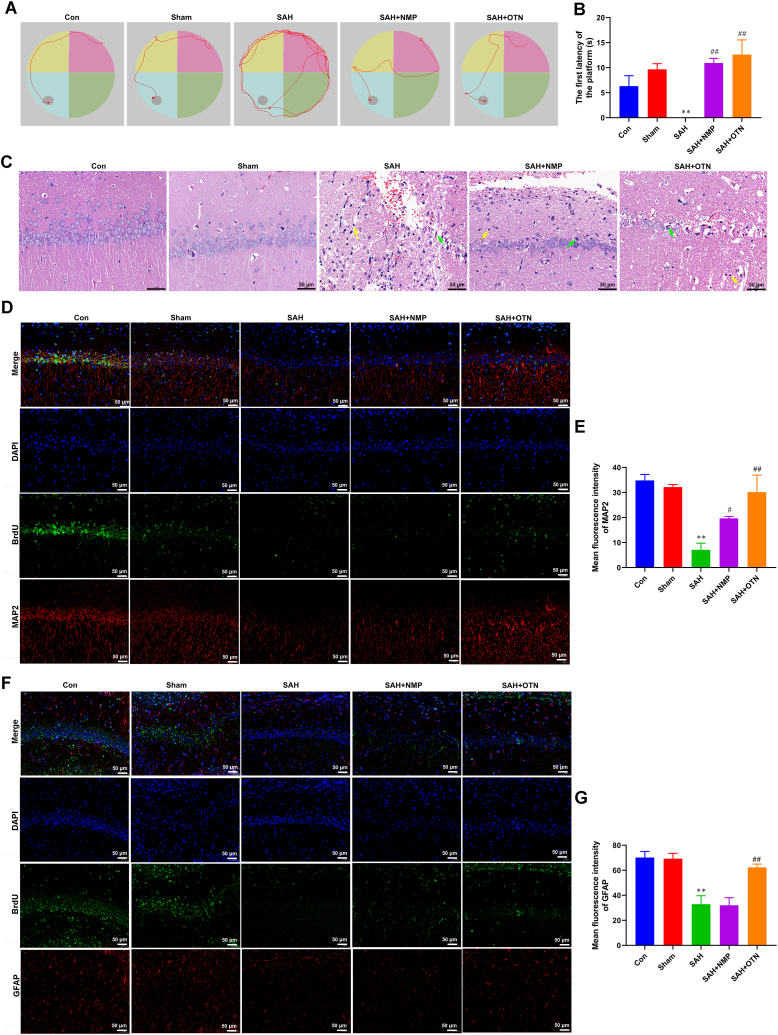
**OTN improved cognitive impairment and hippocampal neuron damage in SAH rats.** (A) Representative tracing graphs in platform trials in Morris water maze. (B) Latency to find the hidden platform in the Morris water maze task. (C) Pathological characteristics of the hippocampus tissues were assessed by H&E stain (Magnification × 400). Yellow arrows, Glial cell hyperplasia. Green arrows, pyramidal cells degeneration. (D and E) Immunofluorescence (IF) double staining of BrdU and MAP2 was used to detect the number of proliferating hippocampal neurons (Magnification × 200). (F and G) IF double staining of BrdU and GFAP was used to detect the number of proliferating hippocampal astrocytes (Magnification × 200). **P < 0.01 vs Sham, ^#^*P* < 0.05 vs SAH, **P < 0.01 vs SAH. n = 6 (Samples from all 6 rats were used in the experiments, and quantitative analyses were also based on data from these 6 rats).

**Fig. 3:**
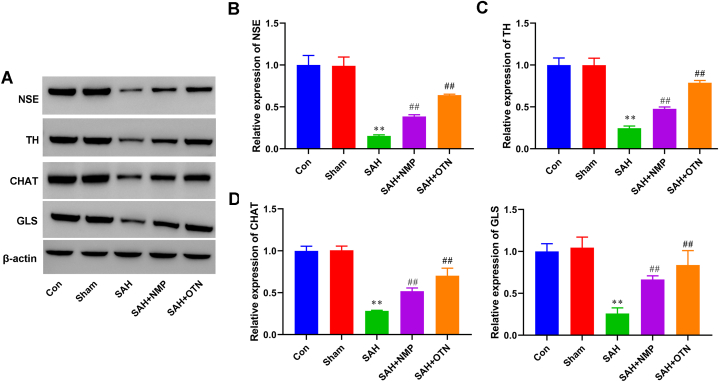
**OTN treatment promoted neuronal activity and differentiation.** Western blot analysis of NSE, TH, CHAT, and GLS the hippocampus tissues. Protein expression was normalized to β-actin. Whole gel imaging is provided in Supplementary Materials, Fig. S1. **P < 0.01 vs Sham, ^#^*P* < 0.05 vs SAH, **P < 0.01 vs SAH. n = 6 (Samples from all 6 rats were used in the experiments, and quantitative analyses were also based on data from these 6 rats).

Morris water maze test suggested that compared with the sham group, spatial cognitive ability, and spatial memory ability were poor in SAH rats (Fig. 2A and B). After OTN or NMP treatment, the spatial cognitive ability and spatial memory ability were effectively improved in SAH (Fig. 2A and B). Furthermore, to evaluate the damage of hippocampal neurons in SAH rats, histopathological changes were examined by HE stain and IF stain. Compared with rats in the sham group, rats in the SAH group showed disturbed hippocampal neuronal arrangement, reduced neuronal numbers, glial cell hyperplasia, and pyramidal cell necrosis (Fig. 2C). However, the rats subjected to OTN or NMP interventions exhibited a significant decrease in damaged neurons and glial cell hyperplasia, suggesting that both kinds of interventions effectively attenuated the damage of hippocampal tissues in SAH rats (Fig. 2C). Hippocampal tissues were stained for IF with antibodies to MAP2 and BrdU to identify neurons (Fig. 1, Fig. 2E). The results suggested that MAP2 expression was significantly decreased in the SAH group, which was enhanced by OTN and NMP treatment (Fig. 2D and E). We performed double IF labeling of GFAP and BrdU to detect astrocyte regeneration (Fig. 2F and G). The IF stain showed a decreased astroglial proliferation in the SAH group compared with the sham group (Fig. 2F and G). In contrast, the hippocampal tissues in the SAH rats from OTN and NMP groups displayed increased astroglial proliferation (Fig. 2F and G). Western blot analysis suggested that OTN and NMP treatment also promoted the expression of NSE, catecholaminergic neurons (TH expression), cholinergic neurons (CHAT expression), and GLS (Fig. 3A–E).

### 3.2 OTN promoted the proliferation of neural stem cells in SAH rats

**Fig. 4:**
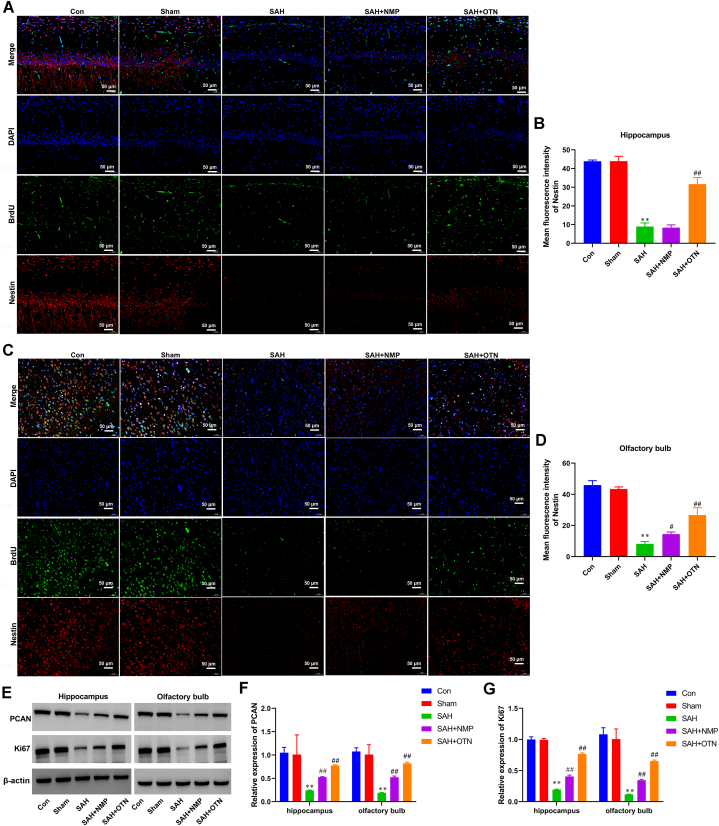
**OTN promoted the proliferation of neural stem cells in SAH rats.** (A and B) IF double staining of BrdU and Nestin was used to detect the number of proliferating neural stem cells in the hippocampus (Magnification × 200). (C and D) IF double staining of BrdU and Nestin was used to detect the number of proliferating neural stem cells in the olfactory bulb (Magnification × 200). (E and G) The expression of PCAN and Ki67 in the hippocampus and olfactory bulb was tested by Western blot. β-actin was used as an internal reference protein. Whole gel imaging is provided in Supplementary Materials, Fig. S2. **P < 0.01 vs Sham, ^#^*P* < 0.05 vs SAH, **P < 0.01 vs SAH. n = 6 (Samples from all 6 rats were used in the experiments, and quantitative analyses were also based on data from these 6 rats).

Next, the proliferation of NSCs in the hippocampus and the olfactory bulb was detected using BrdU/Nestin IF. As shown in Fig. 4A–D, the number of BrdU/Nestin positive cells in the hippocampus and olfactory bulb decreased compared with that in the sham group. OTN and NMP treatment significantly enhanced the proliferation of NSCs in the hippocampus and olfactory after SAH (Fig. 4A–D). Meanwhile, the expression of Ki67 and PCAN were tested by Western blot in the hippocampus and olfactory bulb. Our results further suggested that the expression of Ki67 and PCAN were decreased in the hippocampus and olfactory of SAH rats (Fig. 4E–G). Moreover, OTN and NMP treatment significantly enhanced Ki67 and PCAN expression compared with the SAH group (Fig. 4E–G).

### 3.3 OTN activated Wnt/β-catenin signaling in SAH rats

**Fig. 5:**
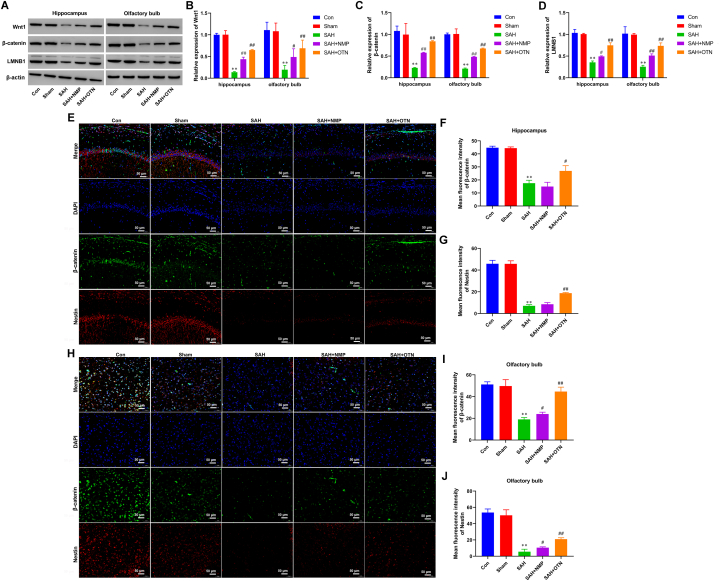
**OTN activated Wnt/β-catenin signaling in SAH rats.** (A–D) The expression of Wnt1, β-Catenin, and LMNB1 in the hippocampus and olfactory bulb was determined by Western blot. β-actin was used as an internal reference protein. Whole gel imaging is provided in Supplementary Materials, Fig. S3. (E–G) Detection of β-catenin expression in hippocampal neural stem cells by IF double-stain of Nestin and β-catenin (Magnification × 200). (H–J) IF double stain of Nestin and β-catenin was used to detect the expression of β-catenin in olfactory bulb neural stem cells (Magnification × 200). **P < 0.01 vs Sham, ^#^*P* < 0.05 vs SAH, **P < 0.01 vs SAH. n = 6 (Samples from all 6 rats were used in the experiments, and quantitative analyses were also based on data from these 6 rats).

Then, we explored the molecular mechanisms by which OTN improved cognitive impairment, abolished neuronal damage, and promoted neural stem cell proliferation in SAH rats. The results indicated that the Wnt/β-catenin signaling pathway was deactivated in SAH rats, which was manifested by decreased expression of Wnt1, β-catenin, and LMNB1 (Fig. 5A–D). As expected, TN and NMP treatment enhanced the expression of Wnt1, β-catenin, and LMNB1 in the hippocampus and olfactory bulb after SAH (Fig. 5A–D). We further examined the colocalization of β-catenin and Nestin by double IF stain (Fig. 5E–J). Our data showed that compared with the sham group, the expression of β-catenin was decreased in the hippocampal and olfactory bulb NSCs, which was recovered by OTN and NMP treatment (Fig. 5E–J).

### 3.4 DKK1 inhibited the OTN-induced activation of the Wnt/β‐catenin signaling pathway in SAH rats

**Fig. 6:**
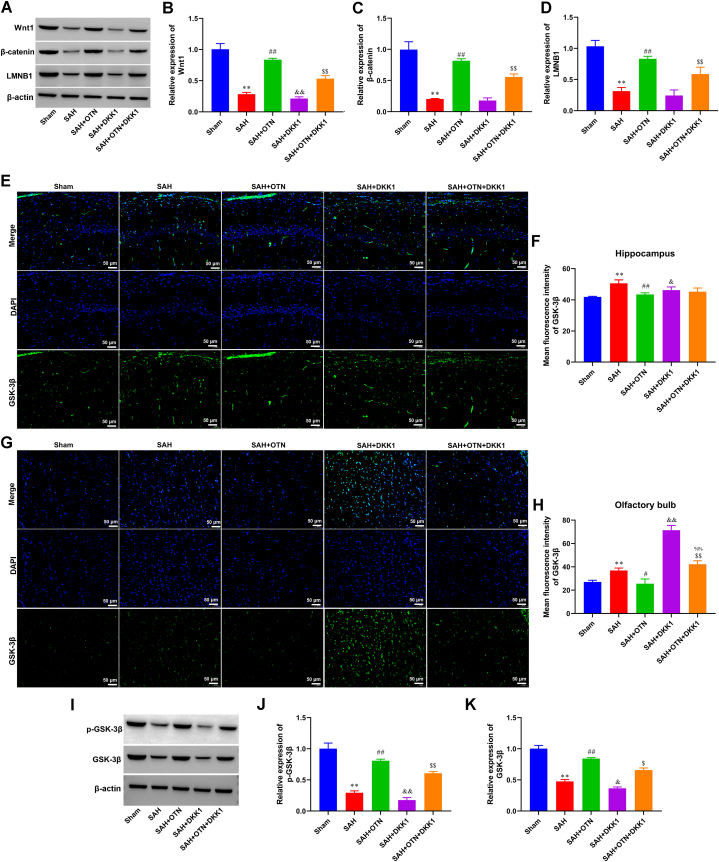
**DKK1 inhibited the OTN-induced activation of the Wnt/β‐catenin signaling pathway in SAH rats.** (A–D) The expression of Wnt1, β-Catenin, and LMNB1 in the hippocampus was assayed by Western blot. β-actin was used as an internal reference protein. Whole gel imaging is provided in Supplementary Materials, Fig. S4. (E and F) IF stain was used to observe the expression of GSK-3β in the hippocampus (Magnification × 200). (G and H) IF stain was used to observe the expression of GSK-3β in the olfactory bulb (Magnification × 200). (I–K) *p*-GSK-3β and GSK-3β protein expression in the hippocampus were detected by Western blot and quantification of Western blot. Whole gel imaging is provided in Supplementary Materials, Fig. S5. **P < 0.01 vs Sham, ^#^*P* < 0.05 vs SAH, ^##^*P* < 0.01 vs SAH, ^$^*P* < 0.05 vs SAH + OTN, ^$$^*P* < 0.01 vs SAH + OTN, ^%%^*P* < 0.01 vs SAH + DKK1. n = 6 (Samples from all 6 rats were used in the experiments, and quantitative analyses were also based on data from these 6 rats).

Western blot analysis showed an up-regulated expression of Wnt1, β-catenin, and LMNB1 in the OTN-treated group, compared with the PAH group (Fig. 6A–D). DKK1 treatment decreased the protein levels of Wnt1, β-Catenin, and LMNB1 (Fig. 6A–D). Additionally, given that the activation of the Wnt/β‐catenin signaling can be achieved by GSK-3β, we tested the expression and activity of GSK-3β. IF and Western blot assayed that OTN treatment promoted GSK-3β and *p*-GSK-3β expression in the hippocampus and olfactory bulb of SAH rats, which was reversed by DKK1 treatment (Fig. 6E–K).

### 3.5 Inhibition of the Wnt/β-catenin signaling pathway reversed the improvement effect of OTN on cognitive impairment and neuronal damage in SAH rats

**Fig. 7:**
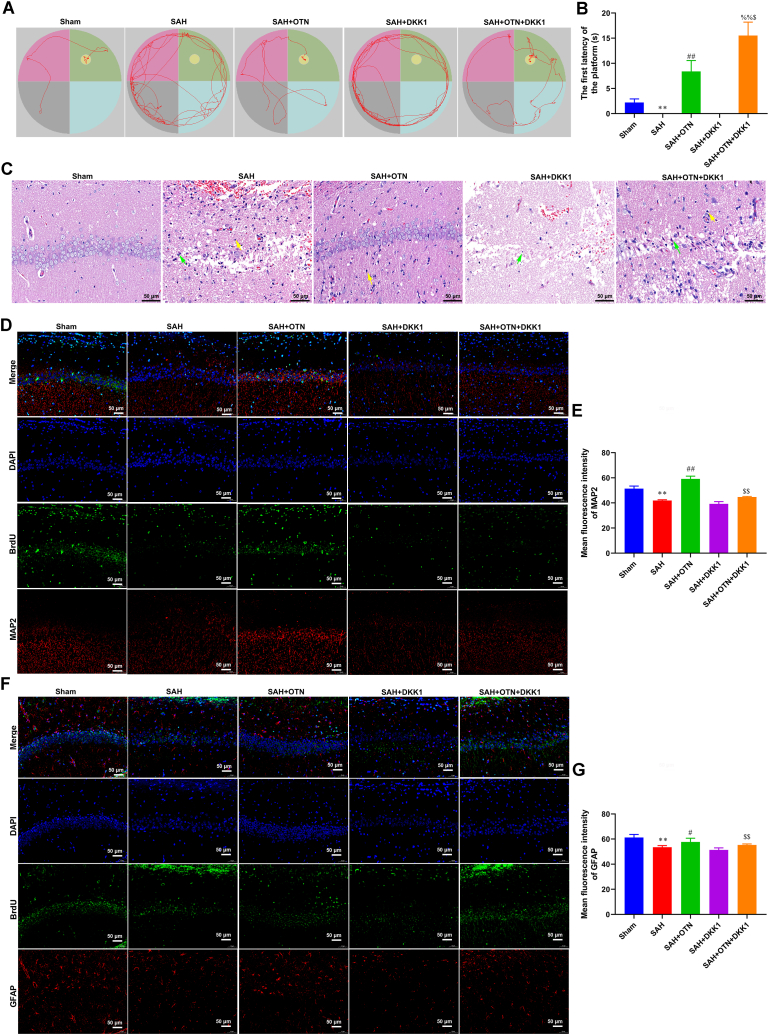
**Inhibition of the Wnt/β-catenin signaling pathway reversed the improvement effect of OTN on cognitive impairment and neuronal damage in SAH rats.** (A) Representative tracing graphs in platform trials in Morris water maze. (B) Latency to find the hidden platform in the Morris water maze task. (C) Pathological characteristics of the hippocampus tissues were assessed by H&E stain (Magnification × 400). Yellow arrows, Glial cell hyperplasia. Green arrows, pyramidal cells degeneration. (D and E) Immunofluorescence (IF) double staining of BrdU and MAP2 was used to detect the number of proliferating hippocampal neurons (Magnification × 200). (F and G) IF double staining of BrdU and GFAP was used to detect the number of proliferating hippocampal astrocytes (Magnification × 200). **P < 0.01 vs Sham, ^#^*P* < 0.05 vs SAH, ^##^*P* < 0.01 vs SAH, ^$^*P* < 0.05 vs SAH + OTN, ^$$^*P* < 0.01 vs SAH + OTN, ^%^*P* < 0.05 vs SAH + DKK1, ^%%^*P* < 0.01 vs SAH + DKK1. n = 6 (Samples from all 6 rats were used in the experiments, and quantitative analyses were also based on data from these 6 rats).

**Fig. 8:**
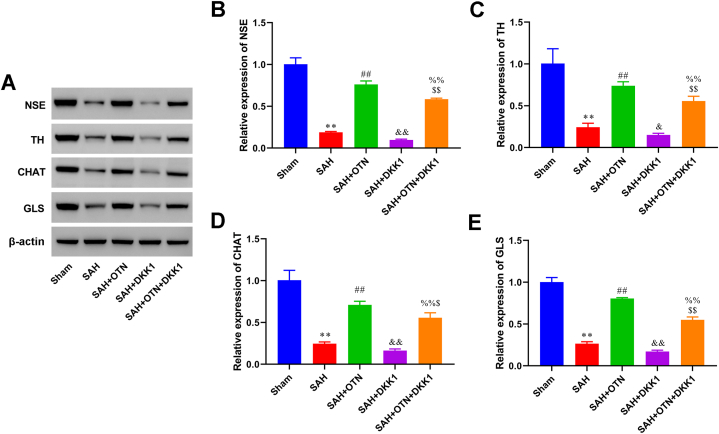
**Inhibition of the Wnt/β-catenin signaling pathway blocked the promoting effects of OTN treatment on neuronal activity and differentiation.** Western blot analysis of NSE, TH, CHAT, and GLS the hippocampus tissues. Protein expression was normalized to β-actin. Whole gel imaging is provided in Supplementary Materials, Fig. S6. **P < 0.01 vs Sham, ^##^*P* < 0.01 vs SAH, ^$^*P* < 0.05 vs SAH + OTN, ^$$^*P* < 0.01 vs SAH + OTN, ^%%^*P* < 0.01 vs SAH + DKK1. n = 6 (Samples from all 6 rats were used in the experiments, and quantitative analyses were also based on data from these 6 rats).

Water maze test results supported that OTN-treated rats had a shorter latency to the platform, which was blocked by DKK1 treatment (Fig. 7A and B). OTN attenuated pathological damage in the hippocampus of PAH rats (Fig. 7C). Furthermore, the hippocampal H&E stain of the OTN + DKK1 group showed a marked increase in neuronal degeneration and glial cell hyperplasia compared with the OTN group (Fig. 7C). OTN group had a significantly increased number of hippocampal neurons and astrocytes in comparison with the PAH group, which were all reversed by DKK1 treatment (Fig. 7D–G). In addition, The expression of neuronal activity and differentiation markers NSE, TH, CHAT, and GLS was increased in the OTN group compared with the SAH group (Fig. 8A–E). Inhibition of Wnt/β-catenin signaling reversed the promotion effect of OTN treatment on neuronal activity and differentiation in PAH rats (Fig. 8A–E).

### 3.6 Inhibition of the Wnt/β-catenin signaling pathway blocked the promoting effect of OTN on the proliferation of NSCs in PAH rats

**Fig. 9:**
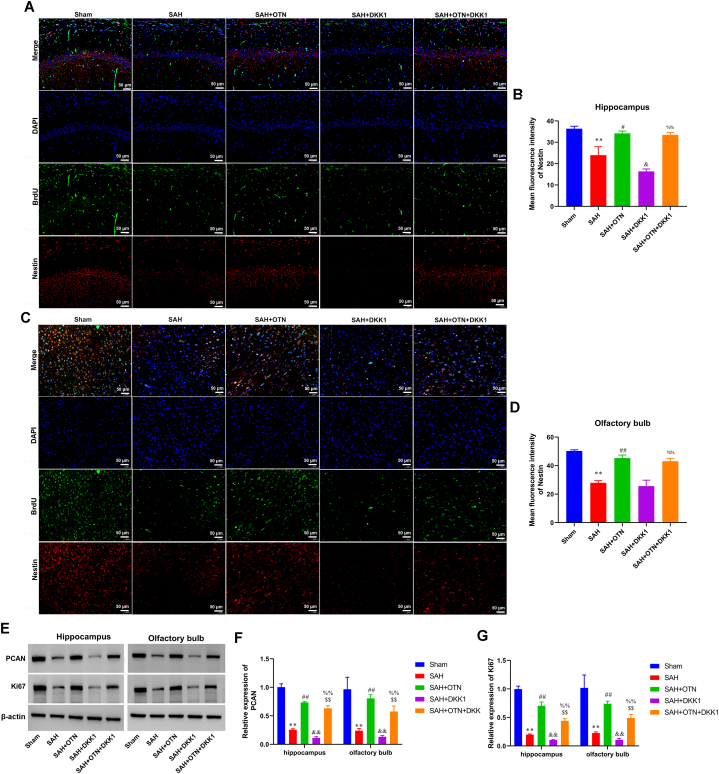
**Inhibition of the Wnt/β-catenin signaling pathway blocked the promoting effect of OTN on the proliferation of NSCs in PAH rats.** (A and B) IF double staining of BrdU and Nestin was used to detect the number of proliferating neural stem cells in the hippocampus (Magnification × 200). (C and D) IF double staining of BrdU and Nestin was used to detect the number of proliferating neural stem cells in the olfactory bulb (Magnification × 200). (E and G) The expression of PCAN and Ki67 in the hippocampus and olfactory bulb was tested by Western blot. β-actin was used as an internal reference protein. Whole gel imaging is provided in Supplementary Materials, Fig. S7. **P < 0.01 vs Sham, ^##^*P* < 0.05 vs SAH, ^##^*P* < 0.01 vs SAH, ^$$^*P* < 0.01 vs SAH + OTN, ^%%^*P* < 0.01 vs SAH + DKK1. n = 6 (Samples from all 6 rats were used in the experiments, and quantitative analyses were also based on data from these 6 rats).

IF results indicated that OTN led to increased NSC proliferation in the hippocampus and olfactory bulb of SAH rats (Fig. 9A–D). Consistently, compared with the DKK1 treatment group, OTN treatment reversed the inhibitory effect of DKK1 on NSC proliferation, as a significant increase in Nestin expression was observed in the hippocampus and olfactory bulb (Fig. 9A–D). To determine proliferation, the expression of PCAN and Ki67 in the hippocampus and the olfactory bulb was tested and found that OTN treatment markedly induced the expression of PCAN and Ki67 in the hippocampus and olfactory bulb after SAH (Fig. 9E–G). Note that DKK1 significantly reduced the expression of PCAN and Ki67 compared with the OTN-treated group (Fig. 9E–G).

## 4 Discussion

Currently, many studies have been devoted to proving the clinical feasibility and efficiency of acupuncture in treating SAH. In a clinically related study, acupuncture has been shown to treat headaches due to SAH [21]. Patients with SAH benefit from electroacupuncture for cerebral vasospasm and functional outcomes [22]. Acupuncture treatment improved functional recovery following SAH and could prevent cerebral vasospasm [23]. These effects may be due to acupuncture modulating the levels of plasma NO and ET-1 to recover endothelial dysfunction [23]. In this study, we found that OTN improved cognitive impairment and hippocampal neuron damage in SAH rats. OTN was previously reported to be employed to treat neurological disorders. It is known that OTN enhanced spatial learning and memory ability in SAMP8 mice by inhibiting the p-p38MAPK expression and the excessive activation of MG to reduce the neuroinflammatory response and neurotoxicity of Aβ and promote synaptic regeneration [18]. OTN inhibited neuro-apoptosis and neuro-inflammation in Alzheimer's disease rats [17]. The possible biological mechanism may be the activated PI3K/AKT/GSK-3β signaling pathway [17]. Furthermore, both OTN and eugenol enhanced learning-memory ability decreased MDA content, and increased SOD and GSH-Px activities in the hippocampus of Alzheimer's disease rats.

Wnt proteins are very important signal molecules in regulating neurodevelopment. In the Wnt signaling pathway, β-catenin is generally phosphorylated in GSK-3β, Axin, and adenomatous polyposis coli (APC) complex, thus entering the ubiquitin/proteasome degradation pathway [24,25]. Wnt, together with Frizzled and LRP 5/6 forming a trimeric complex, activates the intracellular disheveled (Dvl) [26]. Activated Dvl inhibits GSK-3β and then leads to stabilization, accumulation, and further translocation of β-catenin into the nucleus to activate, together with TCF/LEF, the transcription of the target genes, resulting in a series of biological consequences [27,28]. It has been previously demonstrated that HLY78 protected blood-brain barrier integrity through the Wnt/β-catenin signaling pathway following SAH in rats [29]. Moreover, HLY78 attenuated neuronal apoptosis and improved neurological deficits through the LRP6/GSK3β/β-catenin signaling pathway after SAH in rats [30]. GSK-3β is a multifunctional serine/threonine kinase that participates in a variety of signaling pathways and plays a role in promoting apoptosis in different types of cells and various organs such as nerve cells, cardiomyocytes, and tumor cells [31,32]. Recent studies have found that GSK-3β is widely expressed in the cerebral cortex and hippocampus, participates in the control of synaptic plasticity and memory formation and plays an important role in the regulation of cognitive abilities such as learning, memory, and execution [33,34]. Activation of galanin receptor 1 (GalR1) using M617 attenuated neuronal apoptosis through the ERK/GSK-3β/TIP60 pathway after SAH in rats [35]. Methylene blue notably inhibited neuroinflammation after SAH in rats through the Akt/GSK-3β/MEF2D signaling pathway [36]. The administration of COG1410 elevated the expression of the autophagic marker in neurons and simultaneously reversed the neurological deficits after SAH in rats by phosphorylating GSK-3β [37]. In the present study, Wnt/β-catenin/GSK-3β signaling pathway inhibition reversed the improvement effect of OTN on cognitive impairment and neuronal damage in SAH rats and blocked the promotion effect of OTN on NSC proliferation.

## 5 Conclusion

This is the first study to demonstrate the role and potential mechanisms of OTN in the SAH model. OTN improved cognitive impairment, abolished neuronal damage, and promoted neural stem cell proliferation via activating the Wnt/β-catenin/GSK-3β signaling pathway after SAH (Fig. 1B). Thus, OTN may be a promising therapeutic option for patients with SAH.

## Approval of the submission

All authors and responsible authorities where the work was carried out have approved publication.

## Informed consent

Not applicable.

## Data availability statement

Data will be made available on request.

## Funding

This study was supported by a grant from the 10.13039/501100001809National Science Foundation of China (81873387), the National Key Innovation Project of Traditional Chinese Medicine (teaching Letter of Traditional Chinese Medicine (2019) 128), the Xianyang City Young and Middle-aged Science and Technology Leading Talent Cultivation Project (Major Technology Innovation Project) (2019k01-52), the Training Program of Shaanxi University of Traditional Chinese medicine (2017PY13), and the 2023 Science and Technology Innovation Team Project of Shaanxi University of Chinese Medicine (2023-CXTD-02).

## Ethics statement

Animals and experimental protocol were conducted according to the guidelines and ethical standards of the Animal Care and Use Ethics Committees of Shaanxi University of Traditional Chinese Medicine (SUCMDL20180313015).

## CRediT authorship contribution statement

**Feng Zhou:** Writing – review & editing, Writing – original draft, Project administration, Methodology, Investigation, Funding acquisition, Formal analysis, Data curation, Conceptualization. **Zhenzhi Wang:** Visualization, Resources, Investigation, Formal analysis, Data curation. **Kang Xiong:** Supervision, Software, Resources, Formal analysis, Data curation. **Meiling Zhang:** Visualization, Supervision, Project administration, Methodology, Formal analysis. **Qiang Wang:** Visualization, Validation, Supervision, Data curation. **Yuan Wang:** Project administration, Investigation, Formal analysis, Data curation. **Xiong Li:** Visualization, Supervision, Resources, Formal analysis.

## Declaration of competing interest

The authors declare that they have no known competing financial interests or personal relationships that could have appeared to influence the work reported in this paper.
