# Supplementary material for: Olfactory three needle regulates the proliferation of olfactory bulb neural stem cells and ameliorates brain injury after subarachnoid hemorrhage by regulating Wnt/β-catenin signaling
Source: Heliyon. 2024 Mar 25;10(7):e28551. doi: 10.1016/j.heliyon.2024.e28551 (PMC11002047; doi:10.1016/j.heliyon.2024.e28551)
Supplement: Multimedia component 1 [file mmc1.docx]

**Figure S1: The original images of Western blot.**

NSE

1.Con, 2.Sham, 3.SAH, 4.SAH+NMP, 5.SAH+OTN

**
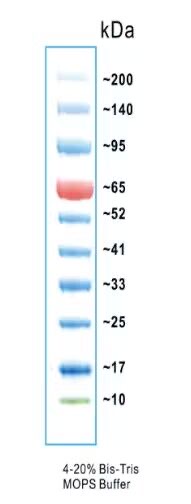

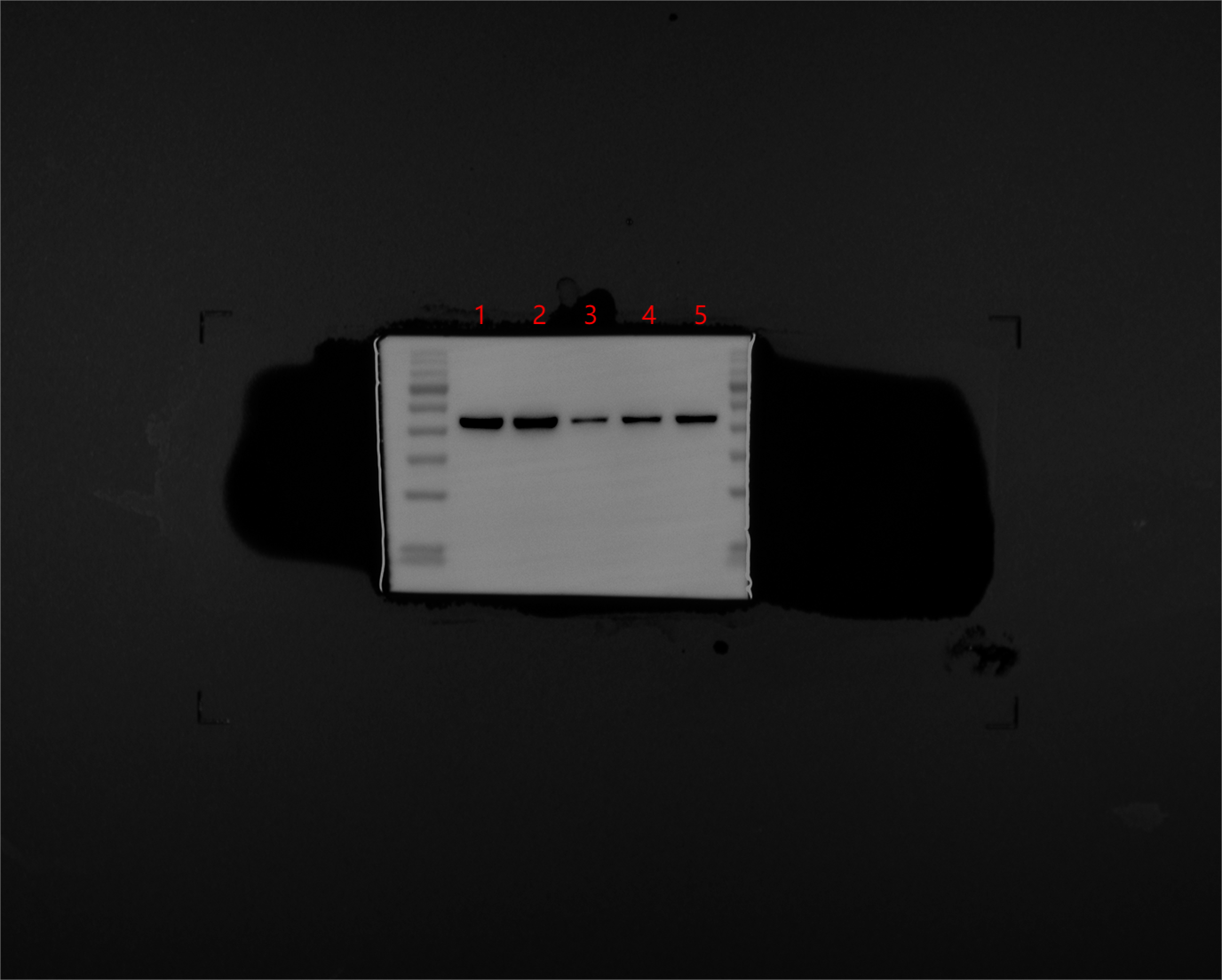
**

TH

1.Con, 2.Sham, 3.SAH, 4.SAH+NMP, 5.SAH+OTN

**
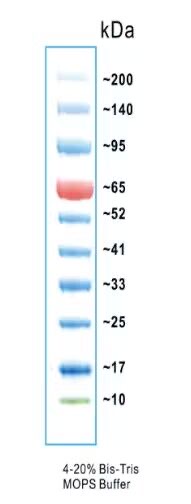

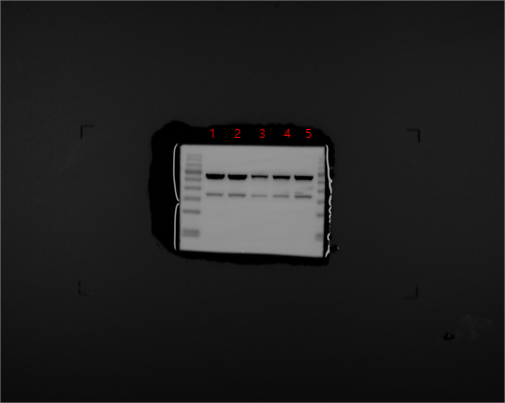
**

CHAT

1.Con, 2.Sham, 3.SAH, 4.SAH+NMP, 5.SAH+OTN

**
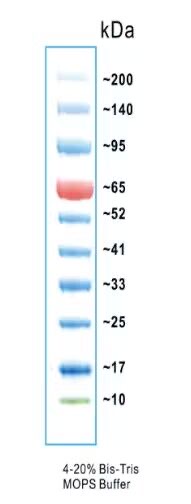

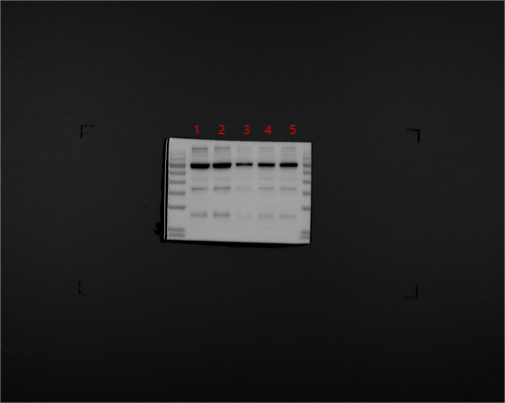
**

GLS

1.Con, 2.Sham, 3.SAH, 4.SAH+NMP, 5.SAH+OTN

**
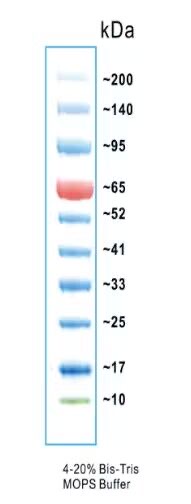

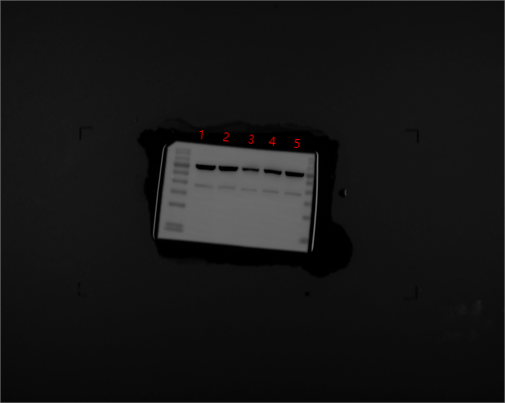
**

β-actin

1.Con, 2.Sham, 3.SAH, 4.SAH+NMP, 5.SAH+OTN

**
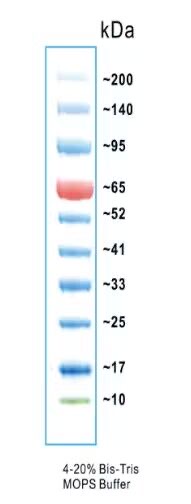

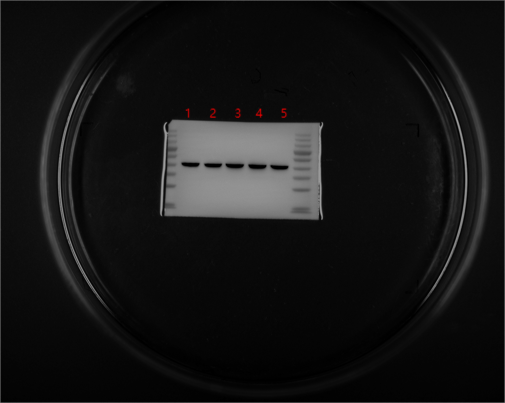
**

**Figure S2: The original images of Western blot.**

Hippocampus: PCAN

1.Con, 2.Sham, 3.SAH, 4.SAH+NMP, 5.SAH+OTN

**
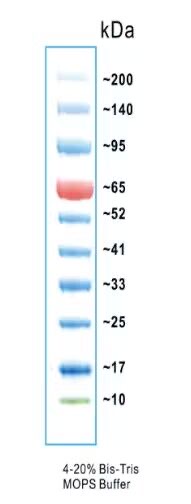

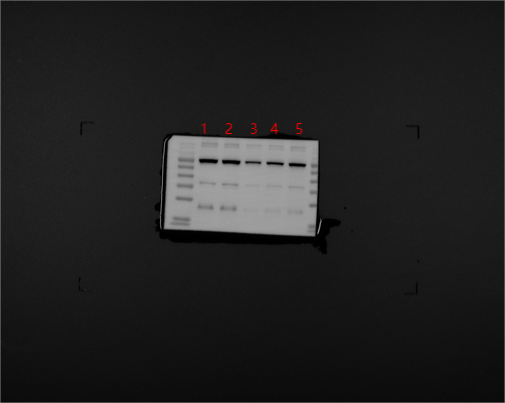
**

Hippocampus: Ki67

1.Con, 2.Sham, 3.SAH, 4.SAH+NMP, 5.SAH+OTN

**
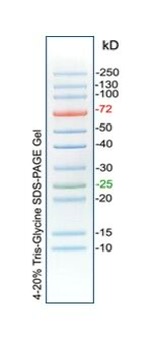

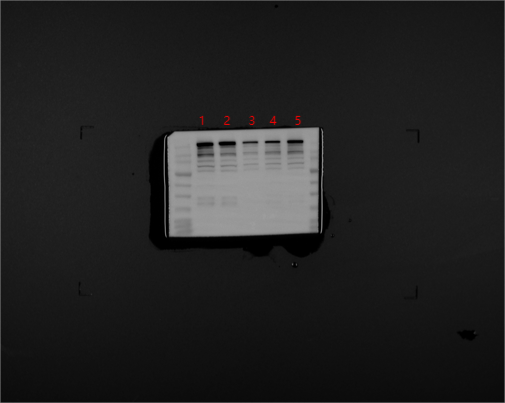
**

Hippocampus: β-actin

1.Con, 2.Sham, 3.SAH, 4.SAH+NMP, 5.SAH+OTN

**
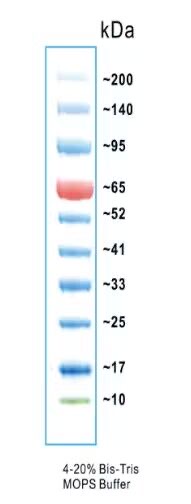

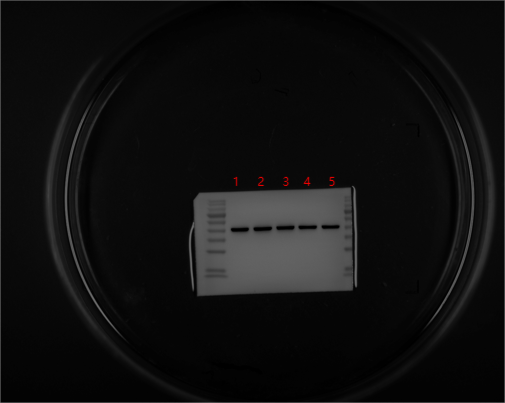
**

Olfactory bulb: PCAN

1.Con, 2.Sham, 3.SAH, 4.SAH+NMP, 5.SAH+OTN

**
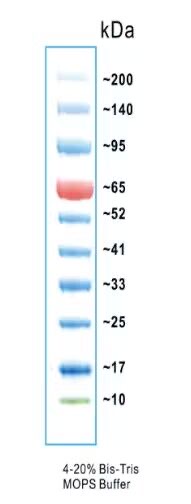

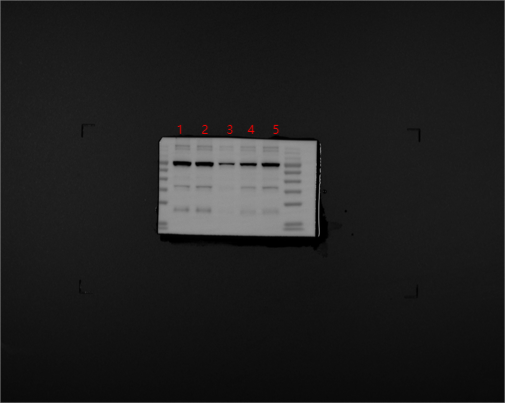
**

Olfactory bulb: Ki67

1.Con, 2.Sham, 3.SAH, 4.SAH+NMP, 5.SAH+OTN

**
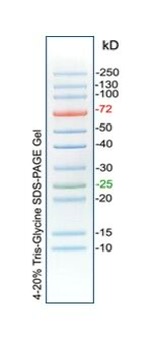

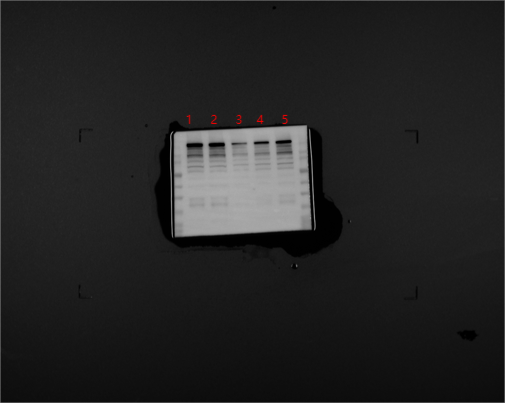
**

Olfactory bulb: β-actin

1.Con, 2.Sham, 3.SAH, 4.SAH+NMP, 5.SAH+OTN

**
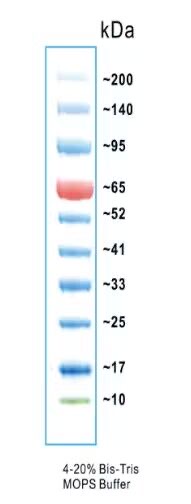

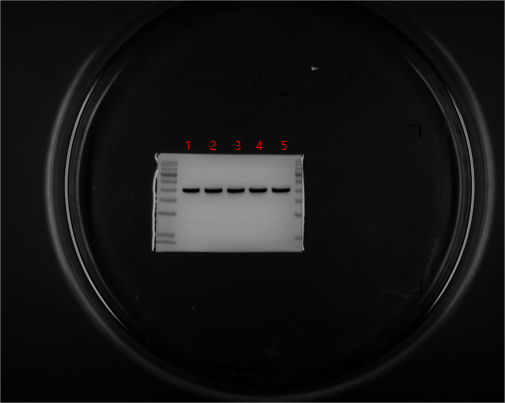
**

**Figure S3****: The original images of Western blot.**

Hippocampus: Wnt1

1.Con, 2.Sham, 3.SAH, 4.SAH+NMP, 5.SAH+OTN

**
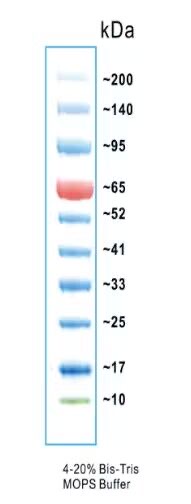

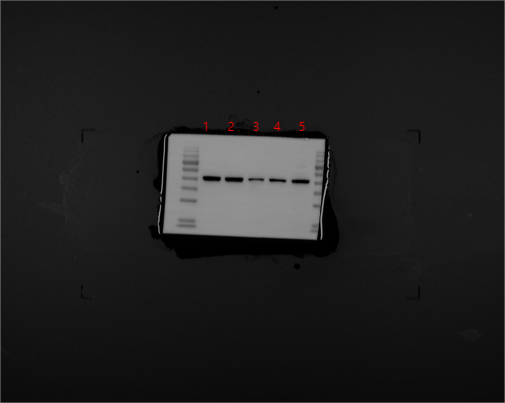
**

Hippocampus: β-catenin

1.Con, 2.Sham, 3.SAH, 4.SAH+NMP, 5.SAH+OTN

**
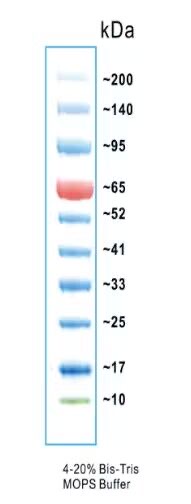

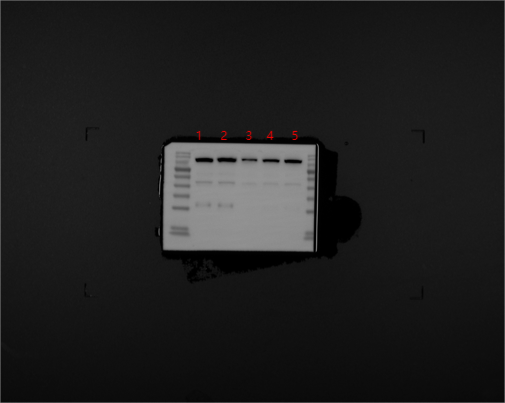
**

Hippocampus: LMNB1

1.Con, 2.Sham, 3.SAH, 4.SAH+NMP, 5.SAH+OTN

**
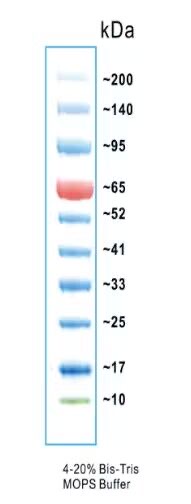

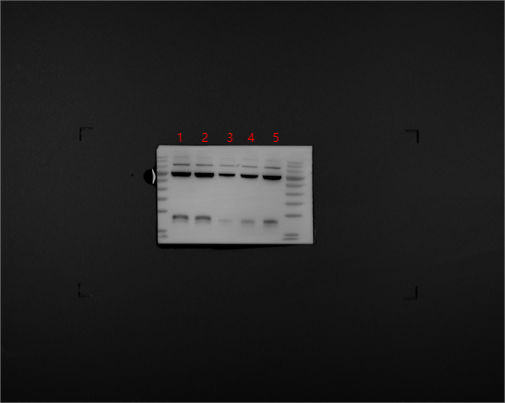
**

Hippocampus: β-actin

1.Con, 2.Sham, 3.SAH, 4.SAH+NMP, 5.SAH+OTN

**
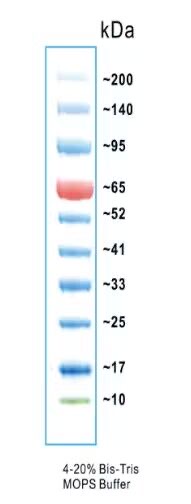

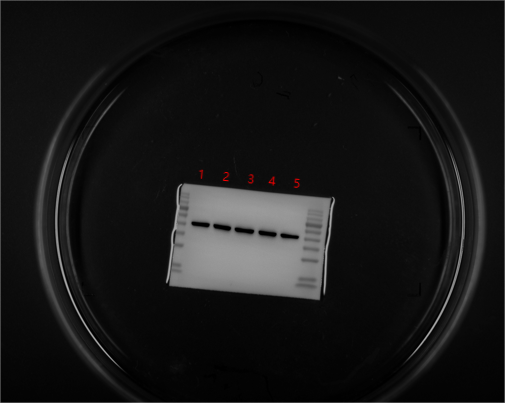
**

Olfactory bulb: Wnt1

1.Con, 2.Sham, 3.SAH, 4.SAH+NMP, 5.SAH+OTN

**
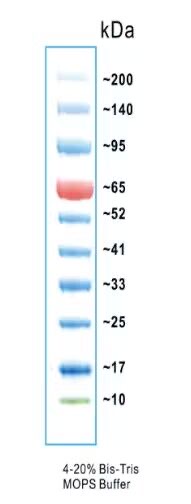

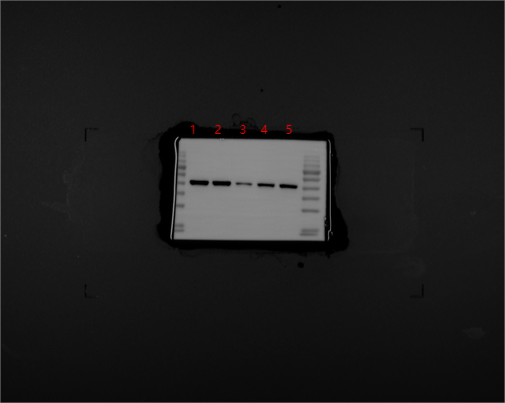
**

Olfactory bulb: β-catenin

1.Con, 2.Sham, 3.SAH, 4.SAH+NMP, 5.SAH+OTN

**
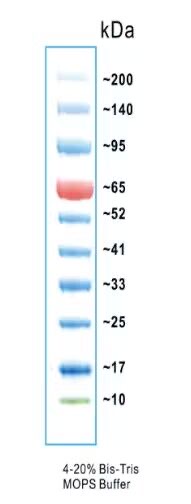

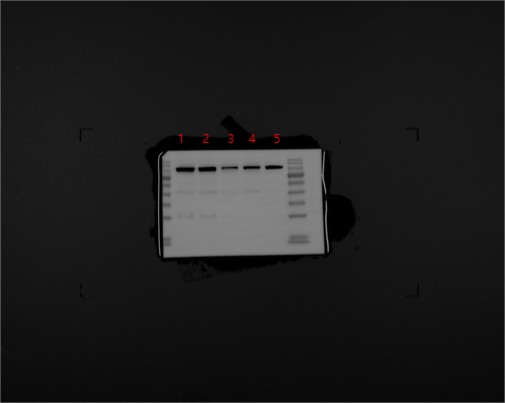
**

Olfactory bulb: LMNB1

1.Con, 2.Sham, 3.SAH, 4.SAH+NMP, 5.SAH+OTN

**
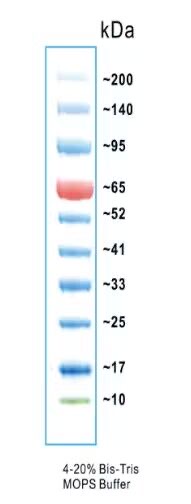

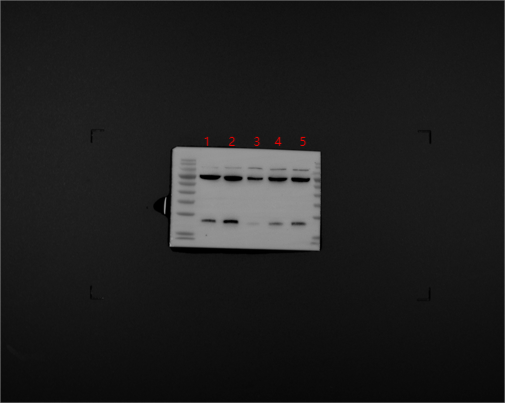
**

Olfactory bulb: β-actin

1.Con, 2.Sham, 3.SAH, 4.SAH+NMP, 5.SAH+OTN

**
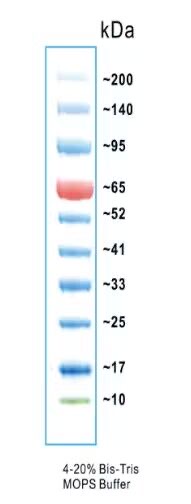

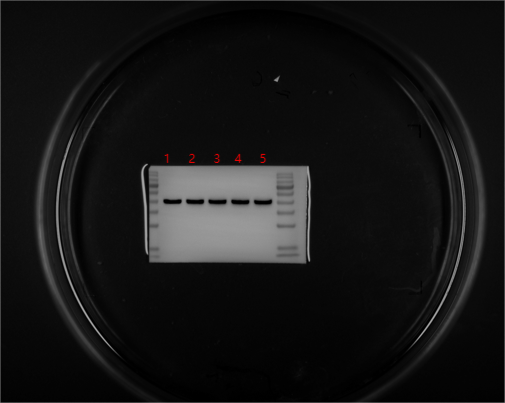
**

**Figure S4: The original images of Western blot.**

Wnt1

1.Sham, 2.SAH, 3.SAH+OTN, 4.SAH+DKK1, 5.SAH+OTN+DKK1

**
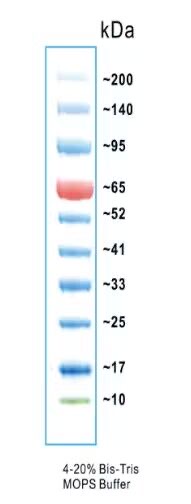

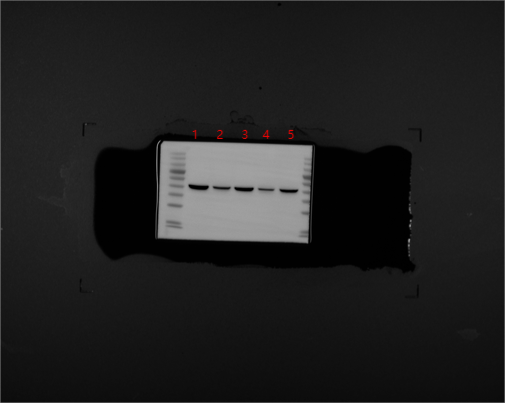
**

β-catenin

1.Sham, 2.SAH, 3.SAH+OTN, 4.SAH+DKK1, 5.SAH+OTN+DKK1

**
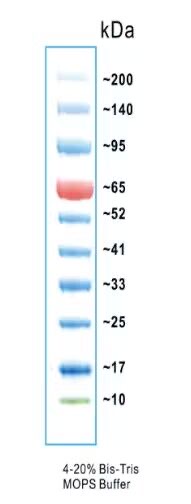

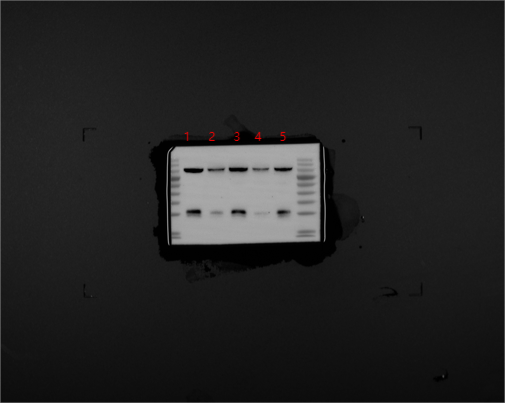
**

LMNB1

1.Sham, 2.SAH, 3.SAH+OTN, 4.SAH+DKK1, 5.SAH+OTN+DKK1

**
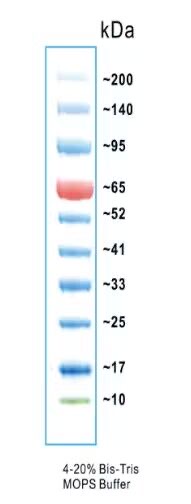

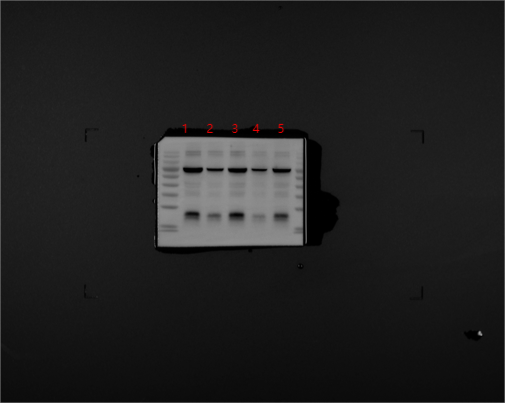
**

β-actin

1.Sham, 2.SAH, 3.SAH+OTN, 4.SAH+DKK1, 5.SAH+OTN+DKK1

**
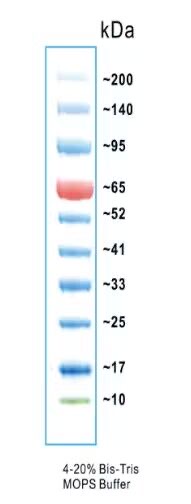

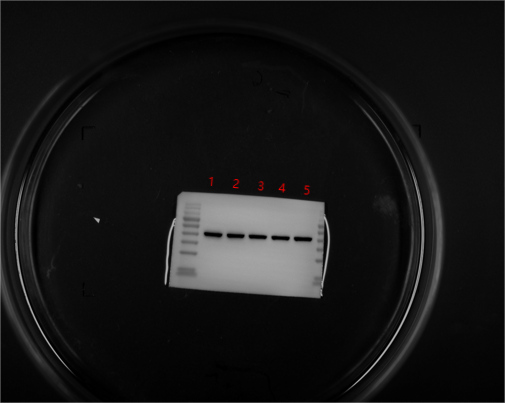
**

**Figure S5: The original images of Western blot.**

p-GSK-3β

1.Sham, 2.SAH, 3.SAH+OTN, 4.SAH+DKK1, 5.SAH+OTN+DKK1

**
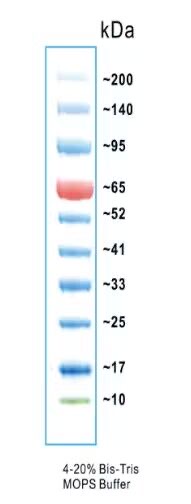

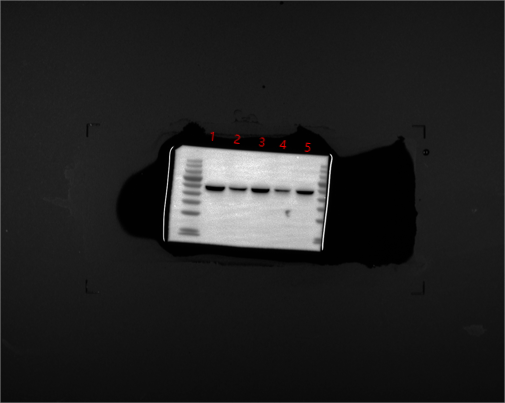
**

GSK-3β

1.Sham, 2.SAH, 3.SAH+OTN, 4.SAH+DKK1, 5.SAH+OTN+DKK1

**
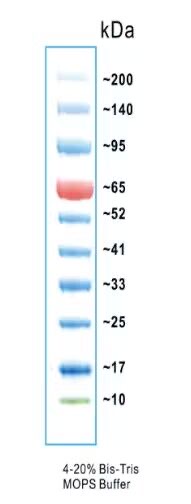

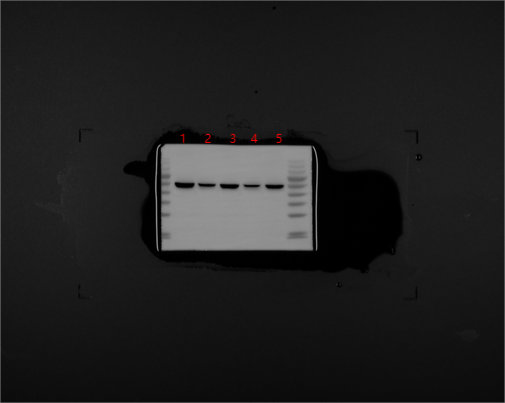
**

β-actin

1.Sham, 2.SAH, 3.SAH+OTN, 4.SAH+DKK1, 5.SAH+OTN+DKK1

**
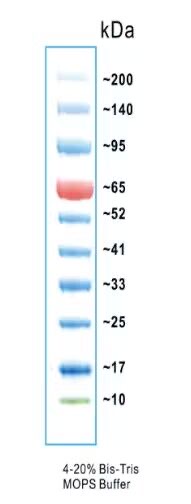

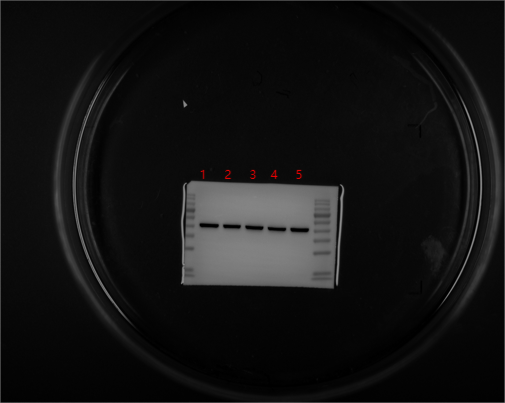
**

**Figure S6: The original images of Western blot.**

NSE

1.Sham, 2.SAH, 3.SAH+OTN, 4.SAH+DKK1, 5.SAH+OTN+DKK1

**
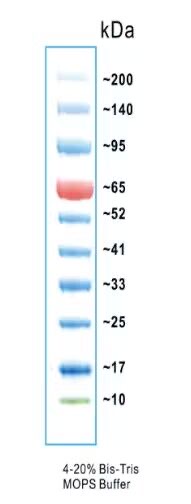

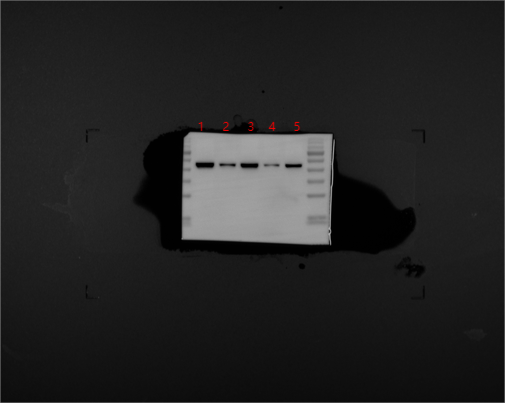
**

TH

1.Sham, 2.SAH, 3.SAH+OTN, 4.SAH+DKK1, 5.SAH+OTN+DKK1

**
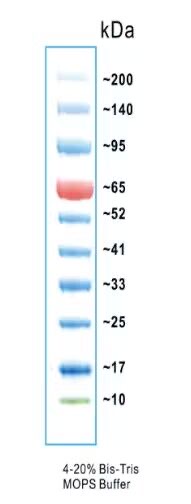

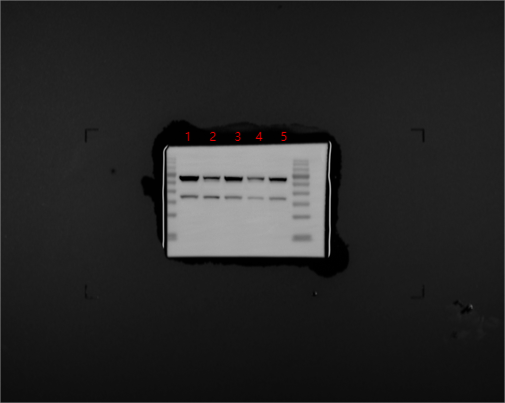
**

CHAT

1.Sham, 2.SAH, 3.SAH+OTN, 4.SAH+DKK1, 5.SAH+OTN+DKK1

**
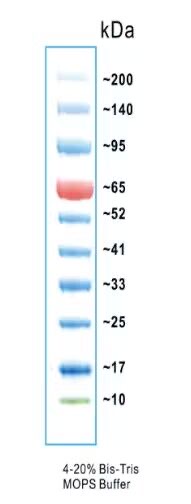

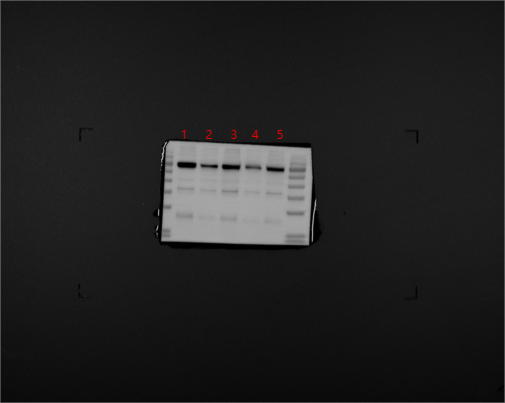
**

GLS

1.Sham, 2.SAH, 3.SAH+OTN, 4.SAH+DKK1, 5.SAH+OTN+DKK1

**
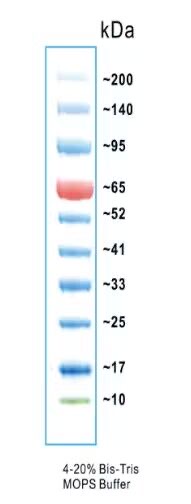

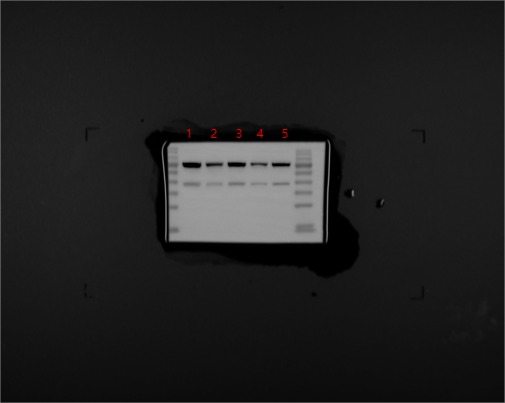
**

β-actin

1.Sham, 2.SAH, 3.SAH+OTN, 4.SAH+DKK1, 5.SAH+OTN+DKK1

**
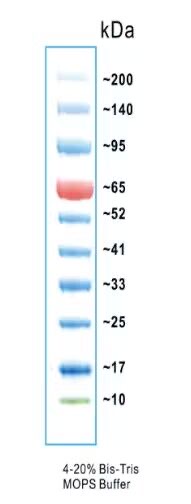

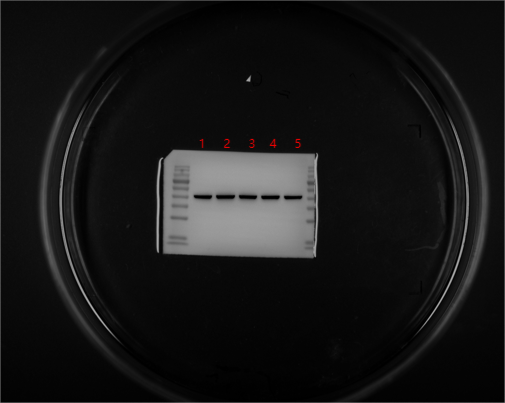
**

**Figure S7: The original images of Western blot.**

Hippocampus: PCAN

1.Sham, 2.SAH, 3.SAH+OTN, 4.SAH+DKK1, 5.SAH+OTN+DKK1

**
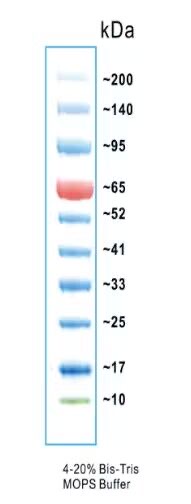

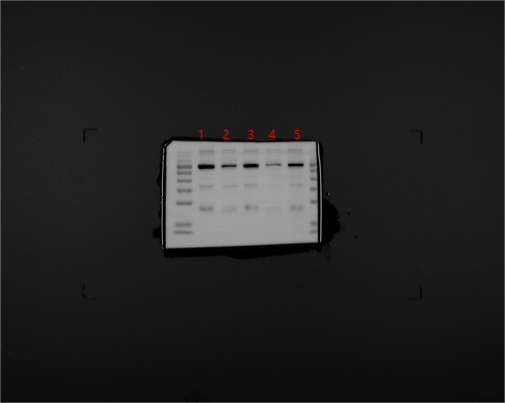
**

Hippocampus: Ki67

1.Sham, 2.SAH, 3.SAH+OTN, 4.SAH+DKK1, 5.SAH+OTN+DKK1

**
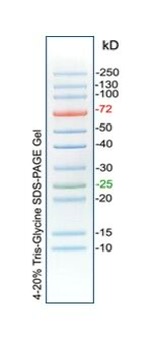

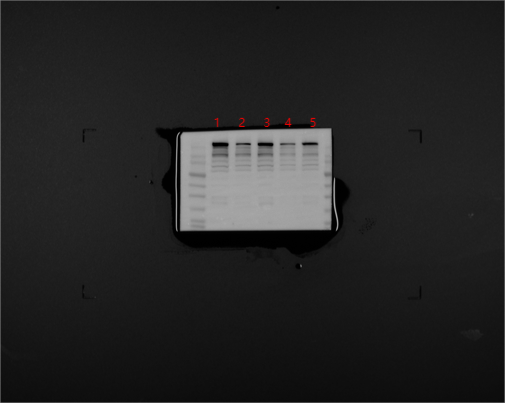
**

Hippocampus: β-actin

1.Sham, 2.SAH, 3.SAH+OTN, 4.SAH+DKK1, 5.SAH+OTN+DKK1

**
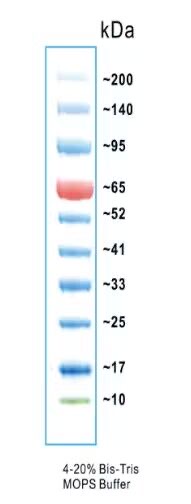

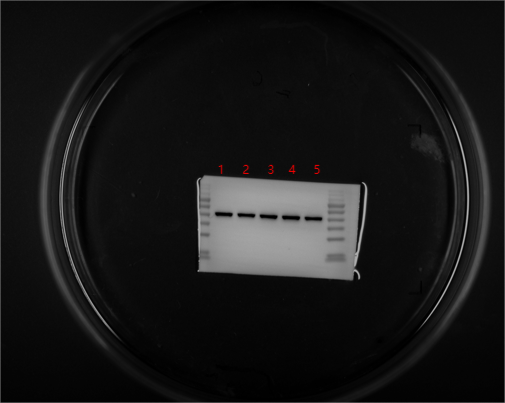
**

Olfactory bulb: PCAN

1.Sham, 2.SAH, 3.SAH+OTN, 4.SAH+DKK1, 5.SAH+OTN+DKK1

**
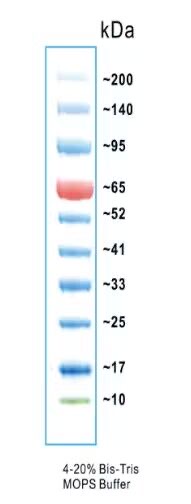

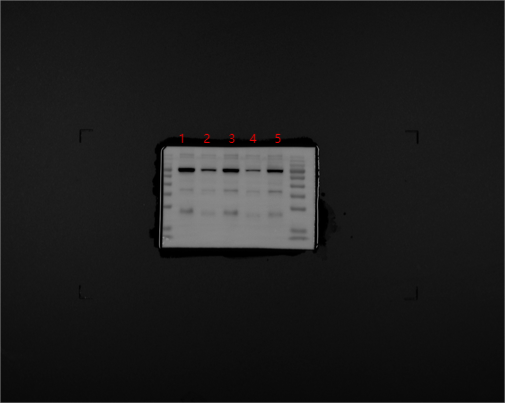
**

Olfactory bulb: Ki67

1.Sham, 2.SAH, 3.SAH+OTN, 4.SAH+DKK1, 5.SAH+OTN+DKK1

**
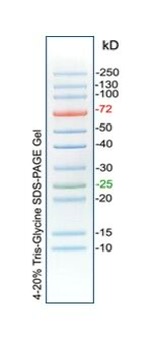

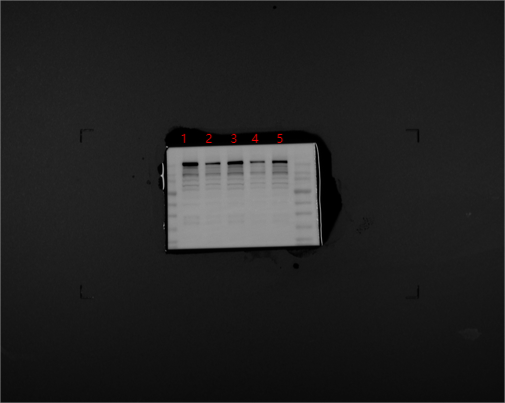
**

Olfactory bulb: β-actin

1.Sham, 2.SAH, 3.SAH+OTN, 4.SAH+DKK1, 5.SAH+OTN+DKK1

**
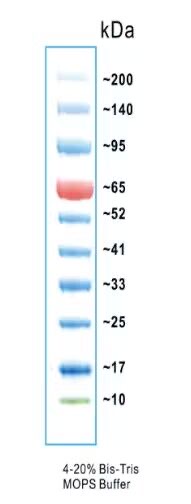

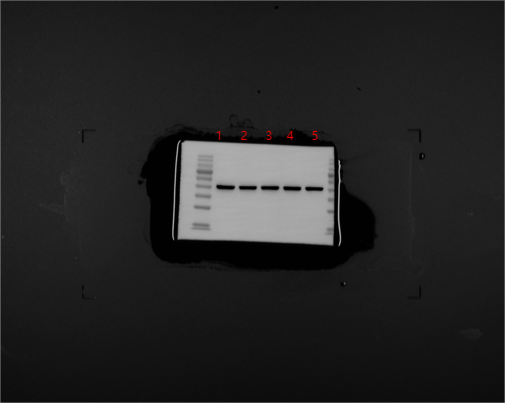
**
